# Lipid Droplets Define a Sub-Population of Breast Cancer Stem Cells

**DOI:** 10.3390/jcm9010087

**Published:** 2019-12-29

**Authors:** Benjamin J. Hershey, Roberta Vazzana, Débora L. Joppi, Kristina M. Havas

**Affiliations:** IFOM Foundation, FIRC Institute of Molecular Oncology, Via Adamello 16, 20139 Milan, Italy; benjamin.hershey@ifom.eu (B.J.H.); Roberta.vazzana@ifom.eu (R.V.);

**Keywords:** cancer stem cells, breast cancer, lipid, metabolism, therapeutic resistance

## Abstract

Tumor recurrence is now the leading cause of breast cancer-related death. These recurrences are believed to arise from residual cancer stem cells that survive initial therapeutic intervention. Therefore, a comprehensive understanding of cancer stem cell biology is needed to generate more effective therapies. Here we investigate the association between dysregulation of lipid metabolism and breast cancer stem cells. Focusing specifically on lipid droplets, we found that the lipid droplet number correlates with stemness in a panel of breast cell lines. Using a flow cytometry-based method developed for this study, we establish a means to isolate cells with augmented lipid droplet loads from total populations and show that they are enriched in cancer stem cells. Furthermore, pharmacological targeting of fatty acid metabolism reveals a metabolic addiction in a subset of cell lines. Our results highlight a key role for the lipid metabolism in the maintenance of the breast cancer stem cell pool, and as such, suggest it as a potential therapeutic target.

## 1. Introduction

Despite advancements in early detection, the World Health Organization reports that over 0.5 million women still succumb to breast cancer every year. The majority of these deaths are attributed to tumor recurrences, which are largely believed to arise from residual cancer stem cells (CSC) that survive the initial therapeutic intervention [1]. According to the CSC model of tumorigenesis, this population of cells is responsible for the origin, progression, and recurrence of the tumor, and therefore, for any therapy to have success, it must be able to effectively target this population [2]. Despite their central role in the development of malignant disease, CSCs remain poorly characterized. The characterization of this population has been hampered, in part, by the lack of robust markers for their identification. Thus, highlighting the necessity to identify more robust markers and therapeutic strategies for CSCs.

Given their central role in promoting tumor progression, the identification and characterization of breast CSCs remain an active area of research. To date, most rely upon the combinatorial expression of cell surface markers such as the cluster of differentiation (CD) 24 and CD44 for the identification of CSC populations [3]. Recently, metabolic stem cell markers, such as cytoplasmic aldehyde dehydrogenase (ALDH) A1 and A3 have been described as markers of adult stem cells and CSC in a number of tissue types [4,5]. Aldehydes are generated by the metabolism of a wide variety of xenobiotic and endobiotic compounds, including alcohols, amino acids, and anticancer drugs, as well as from lipid peroxidation. Aldehyde dehydrogenases function as detoxifying enzymes through their role in metabolizing aldehydes, thereby protecting cells from oxidative and electrophilic stress [6]. The finding that CSCs can, in part, be identified by a metabolic marker has stimulated an interest in the characterization of CSC metabolism.

In order to meet the increased biochemical demands that accompany increased proliferation, metabolic pathways are frequently dysregulated in cancer. The metabolic reprogramming that accompanies cancer onset is now understood to be essential for the pathogenesis of the disease and accordingly has been added to the list of cancer hallmarks [7,8]. Of the metabolic alterations reported in cancer, none is better studied than aerobic glycolysis, the production of lactate from glucose in the presence of oxygen [9,10]. Glycolytic metabolism has been shown to play an important role in supporting stemness in several cancer types. However, it is becoming increasingly clear that in addition to high glycolytic rates, tumorigenesis is supported by multiple aberrant metabolic processes, including dysregulation of lipid metabolism [11].

A coordinated dysregulation of lipid metabolism is observed in nearly all cancer types. In addition to fulfilling the basic requirements of structural lipids for membrane synthesis, lipids play important roles as signaling molecules and contribute significantly to energy homeostasis. As lipid metabolism affects multiple aspects of cellular biology, it is not surprising that alterations in lipid metabolism affect a diverse range of cellular processes including, growth, proliferation, differentiation, and motility.

In order to fulfill their heightened demand for lipids, cancer cells increase their uptake of exogenous fatty acids. This is achieved through increased surface expression of fatty acid translocase CD36. Interestingly, elevated CD36 expression has been reported in metastasis-initiating cell populations and is inversely correlated with survival prognosis [12]. Once taken up by the cells, the free fatty acids can be shuttled into the mitochondria to produce energy equivalents through fatty acid beta-oxidation(FAO). Maintenance of cellular energy stores by FAO has been shown to be fundamental to the survival of CSCs in scarce nutrient environments [13]. Multiple reports have highlighted the importance of increased exogenous fatty acid metabolism in CSCs [14].

In addition to the increased uptake of exogenous lipids, cancer cells also have the unique property of being able to synthesize lipids. Whilst fatty acid biosynthesis is normally restricted to hepatocytes, adipocytes, or mammary epithelium during lactation, several studies have now demonstrated the ability of cancer cells to preform de novo fatty acid biosynthesis [15,16]. Indeed, the expression of several enzymes involved in de novo fatty acid biosynthesis has been implicated in tumorigenesis and the maintenance of stemness in CSCs [17,18].

On a cellular level, excess fatty acids, either exogenous fatty acids taken up by the cells or products of de novo fatty acid biosynthesis, are processed into triacylglycerides and retained in specialized storage organelles called lipid droplets [19]. Lipid droplets are endoplasmic reticulum (ER)-derived organelles that are comprised of a phospholipid monolayer surrounding a core of neutral lipids, primarily triglycerides and sterol esters. They have long been considered to function as a primary store of energy but several recent reports have begun to highlight their role in a diverse range of cellular functions including ER stress, ROS detoxification, and protein dynamics [20].

Our goal in this study is to assess lipid metabolism as a therapeutic target in a panel of breast cancer cell lines. We report that stemness in breast cancer cell lines correlates with lipid droplet number. In line with this, we devised a FACS based strategy to isolate lipid droplet enriched populations and analyzed them for CSC markers. This analysis revealed that lipid droplet^hi^ populations increase CSC markers, as well as increasing mammosphere-forming efficiency. Utilizing an inhibitor of fatty acid biosynthesis, we were able to effectively target the stem cell population in a subset of breast cancer cell lines, thus demonstrating the potential of lipid metabolism targeting compounds as adjuvants to traditional therapies.

## 2. Experimental Section

### 2.1. Cell Lines and Culture Conditions

In this study, the following human breast cancer cell lines were used: BT474, MCF7, T47D, and MDA-MB-231. BT474 cells were purchased from the Leibniz Institute DSMZ-German Collection of Microorganisms and Cell Cultures and maintained in Roswell Park Memorial Institute, RPMI 1640, (Lonza, Basel, Switzerland) supplemented with 5% fetal bovine serum (Sigma-Aldrich, St. Louis, MO, USA), 2 mM L-Glutamine (Euroclone, Milano, Italy), and 10 μg/mL human insulin (Sigma-Aldrich, St. Louis, MO, USA). MCF7, T47D, and MDA-MB 231 were purchased from the National Cancer Institute, Bethesda, MD, USA (NCI) and maintained in RPMI 1640 supplemented with 10% fetal bovine serum and 2 mM L-Glutamine. All the cell lines were cultured at 37 °C in a humidified atmosphere, 5% CO_2_ incubator.

Additionally, two cell lines, representative of normal breast epithelium, HMEC (Cambrex, East Rutherford, NJ, USA) and MCF10a (European Institute of Oncology, Milan, Italy) were used. HMEC cells were cultured in RPMI 1640 with 10% FBS and 2 mM L-glutamine. MCF10a cells were cultured in Dulbecco’s modified Eagle medium/nutrient mixture F-12 (DMEM-Ham’s F12), (Sigma Aldrich, St. Louis, MO, USA) containing: 5% horse serum (Life Technologies, Carlsbad, CA, USA), 10 μg/mL insulin, 20 ng/mL epidermal growth factor (Vinci Biochem, Florence, Italy), 500 ng/mL hydrocortisone (Sigma Aldrich, St. Louis, MO, USA), 100 ng/mL cholera toxin (Sigma Aldrich, St. Louis, MO, USA) and 2 mM L-glutamine.

### 2.2. 5-(Tetradecyloxy)-2-Furoic Acid (TOFA) Treatments

TOFA (Sigma-Aldrich, T6575, St. Louis, MO, USA) was resuspended in two milliliters of Dimethyl sulfoxide (DMSO) and stored at −20 °C. Experiments in which cells were treated with TOFA, unless otherwise specified, were cultured in the presence of 10 μM TOFA in their normal growth media for forty-eight hours prior to initiating measurements or assays.

### 2.3. Growth Curves for TOFA Sensitivity

To assess the effects of TOFA on cell proliferation each cell line was seeded across five, 96-well plates (Costar^TM^, Corning, NY, USA) at the following cell densities, expressed as cells per well: MDA-MB-231 0.5 × 10^4^, MCF7 1 × 10^4^, BT474 and T47D 1.5 × 10^4^. Additional wells were filled with growth media alone to act as plate blanks. All experimental conditions were set up in triplicate. Upon cell adhesion and spreading, a single plate was taken from each set of five plates, fixed and stained.

Fixation was done by removing the culture media and adding 100 μL of 4% paraformaldehyde solution, PFA, (HIMEDIA, Kennett Square, PA, USA) to each well for ten minutes. The PFA was then removed and 100 μL of Crystal Violet solution (Merck KGaA, Darmstadt, Germany) was added to each well. After twenty minutes, all non-cell associated Crystal Violet was removed by washing the wells with water.

Cells in the remaining sets of four plates were treated with either growth media containing 0.13% DMSO or growth media with 10 μM TOFA (Sigma-Aldrich, St. Louis, MO, USA) in DMSO. Every 24 h, for 96 h, a single plate was removed from each set and processed as described above.

Once in the entire time course, five plates in total per cell line were processed and completely dried. One hundred μL of acetic acid glacial was added to each well and incubated for twenty minutes at room temperature. The log optical density (OD) of each experimental well was then measured at a wavelength of 570 nm using a Wallace Victor 3^TM^ multilabel reader (PerkinElmer Inc., Waltham, MA, USA). Reported OD values were generated by subtracting the average of the three experimental wells from the average of the three corresponding plate blank wells.

### 2.4. Fluorescence-Activated Cell Sorting

BODIPY™ 500/510 C_1_, C_12_ (4,4-Difluoro-5-Methyl-4-Bora-3a,4a-Diaza-*s*-Indacene-3-Dodecanoic Acid) (Molecular Probes, Eugene, OR, USA) stock solution, 1 mg/mL, was prepared in ethanol and kept at −20 °C until used. For FACS sorting, the cells were incubated with 1 μg/mL BODIPY™, following an overnight incubation; they were washed twice, trypsinized, resuspended in Phosphate Buffered Saline(PBS) containing 2% FBS and 0.3% Gentamycin and sorted on a MoFlo Astrios cell sorter (Beckman Coulter, Pasadena, CA, USA).

### 2.5. FACS of Fatty Acid Loaded and TOFA Treated Cells

FACS analysis of fatty acid loaded cells was done by culturing cells to 30–40% confluence. The normal growth media was then spiked with 100 μM oleic acid (Sigma-Aldrich, O1383, St. Louis, MO, USA) and 50 μM palmitic acid (Sigma-Aldrich, P5585, St. Louis, MO, USA) for 48 h. Twelve hours prior to FACS, cells were treated with Bodipy at 1 μg/mL. Immediately before FACS, cells were then trypsinized and spun at 1200 rpm for five minutes. The pellet was gently washed with Dulbecco’s Phosphate Buffered Saline (DPBS) and resuspended in DPBS containing 2%, 0.22 μm filtered, FBS. FACS was conducted using Attune NxT (Thermofisher Scientific Inc., Waltham, MA, USA). FACS data depicted represents analysis done on single, 4’,6-diamindino-2-phenylindole(DAPI)-negative cell populations. FlowJo version 10.4.2 (BD Life Sciences, Franklin Lakes, NJ, USA) was used for the analysis.

The same workflow, with the exception of the fatty acid loading step, was used to assess the impact of TOFA treatment on the lipid droplet content of the experimental cell lines.

### 2.6. Nile Red Staining and Lipid Droplets Quantification Analysis

The Nile Red staining was performed as previously reported [21]. Briefly, Nile red (Molecular Probes, Eugene, OR, USA) stock solution was made in DMSO at a concentration of 1 mg/mL. For cellular staining, cells were seeded on poly-lysine coated coverslips and then fixed in paraformaldehyde, 4%, for fifteen minutes. After washing, the cells were incubated for ten minutes in Nile Red, 1 μL of 1 mg/mL Nile Red stock in 10 mL of 150 mM NaCl, protected from light. Nuclei were stained using Dapi, and finally, coverslips were mounted onto glass slides.

Lipid droplet quantification was performed using a Fiji plug-in (developed by Martini E.). Briefly, after manually delineating an Region of Interest (ROI) around the cells, the plugin identified lipid droplets using the ImageJ’s Find Maxima^2^ algorithm on the maximum projection image after background removal (using the rolling ball algorithm^3^) and noise reduction (with a median filter).

### 2.7. RNA Extraction and Quantitative RT-PCR Analysis

In order to perform the RT-PCR analysis, total RNA was first isolated using a trizol-chloroform extraction with TRIzol reagent (Life Technologies, Carlsbad, CA, USA) and chloroform. For the cells collected following cell sorting, RNA was extracted using the RNeasy mini kit (Qiagen, Hilden, Germany). After the extraction, the RNA was quantified by NanoDrop to assess both concentration and quality. Reverse transcription was performed using the SuperScript III reverse transcriptase kit (Invitrogen, Carlsbad, CA, USA). Gene expression was analyzed using the TaqMan gene expression analysis. The samples were amplified with primers for each gene; β-actin was used as the housekeeping gene. The primer assay IDs used in these experiments were: ACTB, Hs99999903_m1; SOX2, Hs01053049_s1; POU5F1, Hs00742896_s1; KLF4, Hs00358836_m1; ALDH1A1, Hs00946916_m1; ALDH1A3, Hs00167476_m1.

### 2.8. Mammosphere Formation Assay from Cell Lines

The primary mammospheres were produced as previously described [22]. The mammosphere media used in this assay was DMEM-F12 (Biowest, Nuaillé, France) supplemented with B27 supplement (Invitrogen, Carlsbad, CA, USA) and EGF 20 ng/mL (Vinci Biochem, Florence, Italy). To prepare non-adherent plates, standard 6-well plates were coated with 1 ml of 1% agarose solution in PBS. To produce the 1% agarose solution, agarose powder was dissolved in PBS and autoclaved.

Briefly, cells were centrifuged at 580× *g* for two minutes and resuspended in 2 mL PBS. To avoid cellular aggregates, a 22 G needle was used to syringe the cell suspension. We found out that 1 × 10^4^ cells/well was a good seeding density for our cell lines. Cells were plated and incubated in a 5% CO_2_ humidified incubator at 37 °C. After five days, all mammospheres larger than 50 μm were counted and the mammosphere formation efficiency (MFE) was calculated using the following formula: mammosphere forming efficiency (%) = (number of mammospheres per well/number of cells seeded per well) × 100.

### 2.9. Assessment of Lipid Droplet Content Using CD44/CD24 Stem Cell Markers

MDA-MB-231 and BT474 cell lines were cultured in 6-well plates (Falcon^®^, Ref no. 353046, Corning, NY, USA). On the night prior to FACS analysis, cells were treated with BODIPY™ 500/510 C_1_, C_12_, as described in Section 2.4. Following incubation with BODIPY^TM^ 500/510 C_1_, C_12,_ the cells were harvested and incubated in 500 μL of a 1× DPBS, 5% BSA, blocking buffer for forty-five minutes at room temperature. The cells were then stained with Alexa Fluor^®^ 647 mouse anti-human CD24 (BD Pharmingen, Material No. 561644, San Jose, CA, USA) and CD44-VioBlue^®^ mouse anti-human CD44 (Miltenyi Biotec, Order No. 130-113-899, Bergisch Gladbach, Germany) for thirty minutes on ice. The antibody concentrations recommended on the accompanying data sheets were used for the stain. Following staining, the cells were pelleted and washed three times with a 1× DPBS, 1% BSA solution, prior to resuspension in a 1% FBS, 1× DPBS solution. The FACS was conducted using the Attune NxT (Thermofisher Scientific Inc., Waltham, MA, USA). FACS data depicted represents analysis done on single, propidium iodide negative, cell population. FlowJo version 10.4.2 (BD Life Sciences, Franklin Lakes, NJ, USA) was used for the analysis.

### 2.10. Fatty Acid Oxidation Assay

MDA-MB-231, MCF7, T47D, and BT474 cell lines were seeded into 96-well plates (Costar^TM^, Corning, NY, USA) at 7 × 10^4^ cells per well and treated with either the vehicle or 10 μM TOFA in DMSO. After approximately twenty hours, the cells were assessed using a fatty acid oxidation assay (Abcam, ab217602, Cambridge, United Kingdom) used in conjunction with an extracellular O_2_ consumption assay (Abcam, ab197243, Cambridge, UK). The protocols accompanying the assays were followed to assess the cell lines after TOFA treatment. Experimental measurements were made using a Wallac Envision^TM^ 2104 multilabel reader (Perkin-Elmer, Waltham, MA, USA), maintained at 37 °C throughout the course of the experiment. Excitation filter, UV (TRF) 340 and emission filter APC665 were used to assess the status of the oxygen-sensing probe used for the assay. Measurements of the oxygen-sensing probe were made every 90 s for one and a half hours.

### 2.11. Transmitted Light and Fluorescence Microscopy

Mammosphere images were acquired with an EVOS FL imaging system (Thermo Fisher Scientific, Inc., Waltham, MA, USA) transmitted light microscope. Fluorescent images were acquired with laser-scanning confocal microscopes: Leica TCS SP5 laser confocal scanner mounted on a Leica DMI 6000B inverted microscope equipped with motorized stage and HCX PL APO 63X/1.4NA oil immersion objective (Leica Mikrosysteme Vertrieb GmbH, Wetzlar, Germany) and Leica TCS SP2 AOBS laser confocal scanner mounted on a Leica DM IRE2 inverted microscope equipped with HCX PL APO 63X/1.4NA oil immersion objective (Leica Mikrosysteme Vertrieb GmbH, Wetzlar, Germany). For the excitation of fluorochromes dyes, 405, 488, 561, and 633 nm laser lines were used on Leica TCS SP5 and Leica TCS SP2 AOBS. The following settings were maintained for fluorescent images acquisition: digital zoom 2.5 and a 1024 × 1024 scan format.

### 2.12. Kaplan-Meier Plotter

Kaplan–Meier plots were generated using the Kaplan–Meier plotter found at http://kmplot.com/analysis/index.php?p=background [23]. This is an online platform that enables the user to assess the effect of 54,000 genes on survival in 21 cancer types. Prognostic values for PLIN2 mRNA (Affymetrix ID 209122_at) expression was evaluated for a cohort of 3951 breast cancer patients.

### 2.13. Statistical Analysis

All experiments were carried out at least three times unless otherwise indicated. Data were analyzed using GraphPad Prism version 8 statistical software (GraphPad Software, San Diego, CA, USA). Experimental results are reported as mean and standard deviation unless otherwise stated.

## 3. Results

### 3.1. Lipid Droplet Marker PLIN2 Expression Correlates with Disease Progression and Lipid Droplet Number

**Figure 1:**
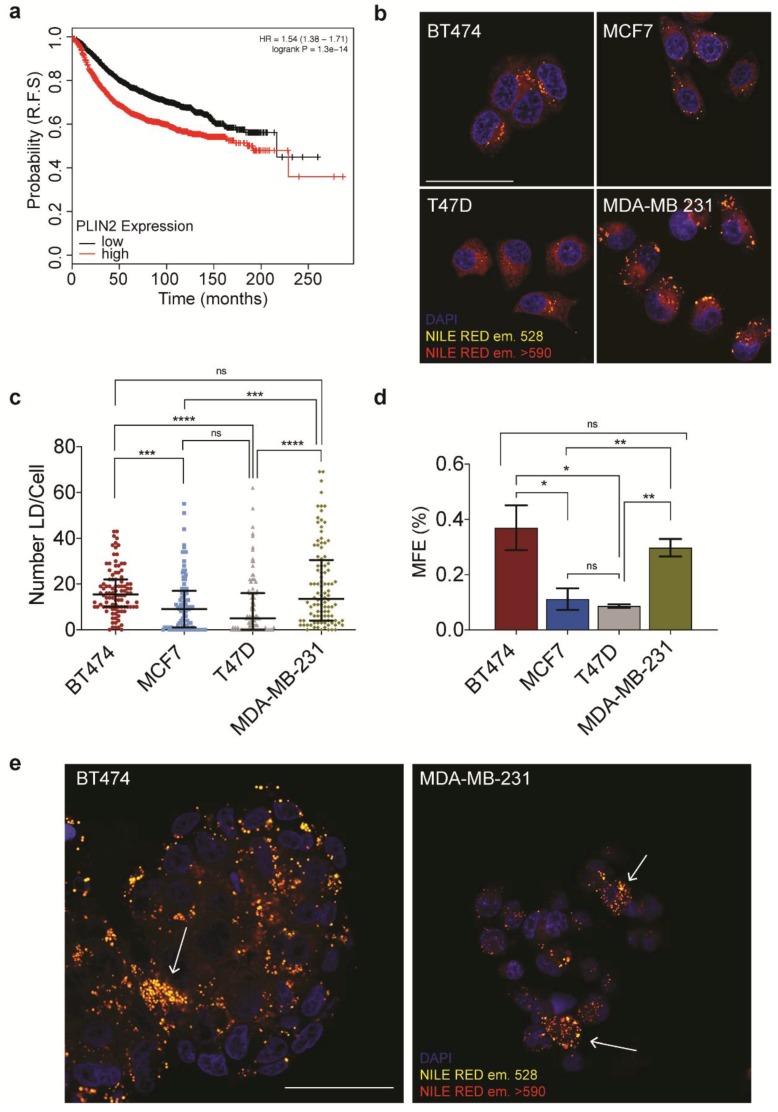
Lipid droplets correlate with breast cancer progression and stemness. (**a**) The effect of PLIN2 expression on relapse-free survival in breast cancer (**b**) Confocal imaging of Nile Red stained BT474, MCF7, T47D, and MDA-MB-231. The scale bar represents 50 μm. (**c**) Quantification of lipid droplets number/cell for 100 cells per cell line (right). (**d**) Mammosphere formation efficiency for the four cell lines used in this study. (**e**) Confocal imaging of mammospheres stained with Nile Red, arrows indicate lipid droplet enriched cells, scale bar represents 50 μm. Data are represented as mean ± SEM. Significance was calculated using two-tailed *t*-tests. * *p* < 0.05, ** *p* < 0.01, *** *p* < 0.001, **** *p* < 0.0001.

An increase in lipid droplet metabolism in therapeutic resistant cell populations has now been reported for several solid tumors, including breast [24,25]. This led us to ask whether there was a correlation between lipid droplets and disease progression in breast cancer. As a proxy for lipid droplets, we chose to use a member of the perilipin family, Perilipin 2 (PLIN2), a lipid-droplet associated structural protein. The over-expression of PLIN2 has previously been reported for several cancer types, although its prognostic value is not clear. Therefore, we asked whether PLIN2 over-expression has any prognostic value in breast cancer. Querying the Kaplan–Meier plotter [23], we have found a significant negative correlation between PLIN2 expression and relapse-free survival in breast cancer patients (Figure 1a). After stratifying for intrinsic breast cancer sub-type, we observed that the negative correlation was maintained in basal, luminal A and luminal B subtypes, although interestingly not in Her2+ breast cancers (Figure S1a).

Next, we set out to investigate whether PLIN2 expression correlates with an increase in lipid droplet number across a panel of breast cancer cell lines. Cell lines were selected to represent the major intrinsic subtypes as classified by their immuno-histological features are represented in Table 1.

We utilized 9-diethylamino-5H-benzo [alpha] phenoxazine-5-one (Nile Red) to assess the lipid droplet content across the panel (Figure 1b). Quantification of the baseline lipid droplet numbers in these cells revealed that although all breast cancer cell lines queried contain lipid droplets, BT474, and MB-MDA-231 had a significantly higher number of lipid droplets in comparison to the other cell lines tested (Figure 1c). Of note, we also quantified lipid droplet number in two control cell lines, MCF10a and HMECs. Despite being models of “healthy mammary epithelium,” both cell lines had an elevated number of lipid droplets in comparison to the cancer cell lines (Figure S1b).

It has yet to be determined whether the increase in lipid droplets that is associated with therapeutic resistance is a consequence of a stress response or if it represents an enrichment of a therapy-resistant sub-clonal population. Considering the CSC theory of therapeutic resistance, we were prompted to ask whether there could be a correlation between lipid droplets and stemness. We began by evaluating the expression of stemness markers utilizing quantitative PCR. We found that the baseline, normalized values, of markers previously published to associated with CSCs including Octamer-binding transcription factor 4 (POUF51), SRY(Sex-determining region Y)-box2 (SOX2), ALDH1A3, and Kruppel -like factor 4 (KLF4) did not show a significant variation in between the cell lines tested (Figure S1c). Notably, the expression of ALDH1A1, which was also included in the panel, was limited to BT474. Breast CSCs can be enriched by culturing in non-adherent, non-differentiating culture conditions, where they grow as small clusters of CSC derived cells referred to as mammospheres. To ask whether there were discernible differences in CSC pools between the four cell lines, we performed mammosphere formation efficiency assays. Despite similar overall levels in the expression of stem cell markers, we observed clear variations in mammosphere formation efficiency between the four cell lines (Figure 1d and Figure S1d).

The mammosphere formation assay suggested a correlation between lipid droplet number and stemness, but we now wanted to demonstrate that lipid droplet enriched populations are indeed found in mammospheres. Therefore we passaged mammospheres produced from MDA-MB-231 and BT474 for two generations and then visualized the lipid droplet containing cells using Nile Red. Interestingly, we found a sub-population of cells within the mammospheres to be highly enriched in lipid droplets (Figure 1e and Figure S1e). Intriguingly, taken together, these observations suggested a link between lipid droplet load and stemness in breast cancer-derived cell lines.

### 3.2. Lipid Droplets Increase Cell Complexity and Provide a Means to Isolate Individual Populations

These observations led us to ask whether CSCs were enriched in the population of cells harboring high numbers of lipid droplets. To address this question, we needed a means to separate lipid droplet-enriched from lipid droplet-depleted populations. Having shown that the accumulation of lipid droplets within the cell is tractable through the use of lipid droplet specific dye Nile Red, we reasoned that we could utilize cell fluorescent fatty acid analogs to sort lipid droplet^hi^ populations from lipid droplet^lo^ populations.

**Figure 2:**
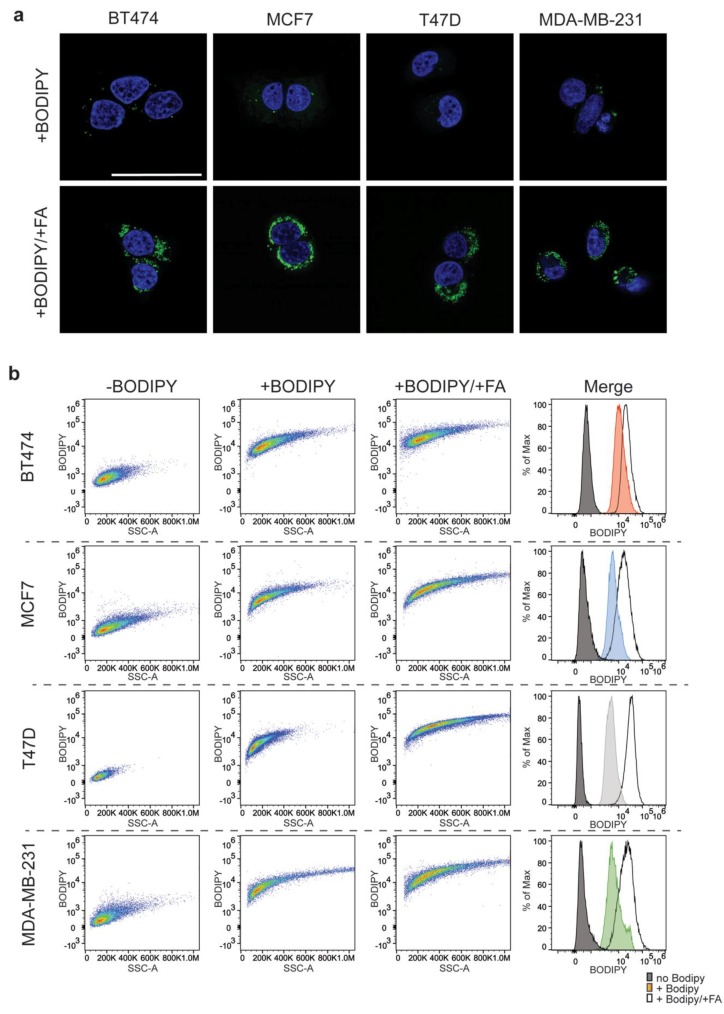
BODIPY^TM^ based FACS protocol distinguishes lipid droplet enriched cells. (**a**) Confocal imaging of BODIPY^TM^ 500,510 loaded cells. Upper panels are representative images from each of the four cell lines following a 12 h incubation with BODIPY^TM^, lower panel representative image of cells incubated with palmitic acid, oleic acid and BODIPY^TM^. The scale bar represents 50 μm. (**b**) Representative plots of BODIPY^TM^ signal in cells, – BODIPY^TM^, + BODIPY^TM,^ and fatty acid loaded cells + BODIPY^TM^. The corresponding histograms represent the FITC (BODIPY^TM^) signal for each condition.

In order to develop a florescenence-activated cell sorting (FACS) based approach for cell sorting, we needed a live cell marker of lipid droplets; we chose to use BODIPY™ 500/510(BODIPY™). BODIPY™ is a fluorescent fatty acid analog that can be used as a marker of lipid droplets and membranes [28]. To understand whether BODIPY^TM^ would be suitable for a FACS based assay, we incubated cells in growth media containing BODIPY™ and evaluated the resulting fluorescence signal using confocal microscopy. Image analysis confirmed that the BODIPY™ was being taken up by the cells and incorporated into the neutral lipid species (triglycerides or sterol esters) that are stored in cytoplasmic lipid droplets. Additionally, membrane incorporation, whereas visible, was not the major contributor to the signal (Figure 2a).

We then proceeded to test whether we could use a FACS-based strategy to identify lipid droplet containing populations using BODIPY™. FACS-based analyses of the BODIPY™ loaded cells versus non-loaded controls revealed a strong shift in the fluorescence signal following excitation at 488 nm for all cell lines (Figure 2b). In order to demonstrate that the BODIPY™ signal being read by the FACS is attributable to the lipid droplet concentration, we tested the system by pre-incubating the cells in media containing additional fatty acids. The addition of exogenous fatty acids to the media resulted in their uptake and metabolism leading to a dramatic increase in lipid droplet number (Figure S2a).

Accordingly, we observed not only a significant increase in BODIPY™ signal in cells that had been pre-loaded with fatty acids but also a marked shift in the side scatter (SSC) measurements of the cells (Figure 2b). SSC at FACS is used as an indicator of the internal complexity or granularity of the cell. These parameters are often used to measure internal cellular components such as granules. Interestingly, increasing the number of lipid droplets also resulted in an increase in the SSC measurements for the treated population. Therefore, like granules, lipid droplets increase the complexity of the cytosol, in a way that was distinguishable at FACS. Taken together, the BODIPY™ driven FACS based strategy provided a robust method to stratify cells based on lipid droplet content.

### 3.3. Lipid Droplets Correlate with Stemness in a Subset of Breast Cancers

**Figure 3:**
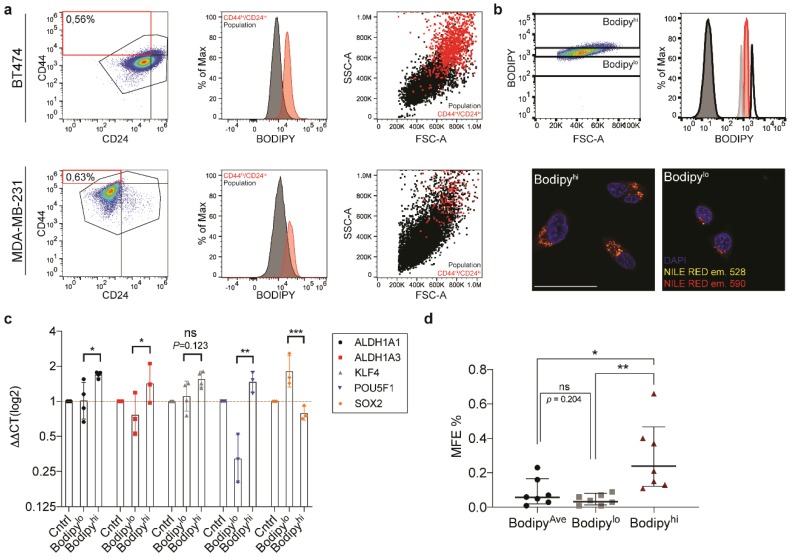
Lipid droplet^hi^ populations are enriched for stem cell markers in BT474 (**a**) FACS-based analysis of CD44^hi^/CD24^lo^ populations in BT474 and MDA-MB-231. Scatter plot showing the distribution of the two surface markers (left), the histogram of MFI for BODIPY^TM^ in CD44^hi^/CD24^lo^ (red) versus total population (black; middle), the dot plot to highlight SSC distribution of CD44^hi^/CD24^lo^ (red) versus total population (black). (**b**) Scatter plot for FITC (BODIPY^TM^) versus FSC illustrating the gating strategy for FACS sort, with histogram for FITC (BODIPY^TM^) levels in sorted BT474 populations. Confocal microscopy of Nile Red stained sorted populations. The scale bar represents 50 μm. (**c**) Quantitative PCR to analyze the expression of a panel of genes associated with stemness for the three sorted populations from BT474. Data are represented as geometric mean ± SD (**d**) Mammosphere formation efficiency for the three sorted populations from BT474 (*n* = 7). Data are represented as geometric mean ± SD. Significance was calculated using the two-tailed *t*-test. * *p* < 0.05, ** *p* < 0.01, *** *p* < 0.001.

The development of FACS based protocols for the identification and isolation of stem cells has been instrumental in shaping our understanding of their contribution to development and pathogenesis. In the breast, the cell population distinguished by CD24^lo/−^/CD44^hi^ has been previously shown to be highly enriched in CSCs both in primary patient material as well as in established breast cancer cell lines [3,29]. The establishment of the BODIPY^TM^ based FACS protocol described above now allowed us to investigate whether CSCs populations, defined by CD24/CD44 levels, contain high levels of lipid droplets. In agreement with our previous data, we observed a 2.5–2.7 fold increase BODIPY^TM^ intensity in the stem enriched CD24^lo/−^/CD44^hi^ pool (Figure 3a and Figure S3). In addition to the observed increase in the BODIPY^TM^ signal, we also detected increased cytosolic complexity (SSC) in the CD24^lo/−^/CD44^hi^ population. This is in accordance with the previous experiments that demonstrated that increasing lipid droplet load led to an increase in SSC. These results further strengthened the earlier observation that lipid droplet number correlates with stemness.

Having established multiple correlations between lipid droplet number and stemness features, we now wanted to demonstrate that populations of cells containing higher lipid droplet numbers were indeed enriched in CSCs. In order to isolate lipid droplet enriched populations from the total population, we applied the FACS based strategy described above. BT474 was chosen due to its propensity to show enrichment in lipid droplet number and mammosphere-forming efficiency. Three separate bins were created to enable sorting of the top (lipid droplet^hi^) 5% and bottom (lipid droplet^lo^) 5% of cells based upon the BODIPY™ fluorescence intensity. Cells were additionally collected from the average (lipid droplet^ave^) BODIPY™ population to serve as a control group (Figure 3b). Following sorting, cells were assessed for lipid droplet number by fluorescence microscopy. Nile Red was again employed to visualize lipid droplets in the sorted cells. The staining confirmed enrichment of lipid droplets in the lipid droplet^hi^ population.

We proceeded to characterize the three populations isolated from the BT474 cell line for stemness traits. Quantitative PCR was performed for a panel of CSC markers. Interestingly, with the exception of SOX2, we saw a marked increase across all stem cell markers in the lipid droplet^hi^ population (Figure 3c), suggesting that the lipid droplet^hi^ population of BT474 was enriched for CSCs. Next, we tested the three isolated populations in mammosphere formation assays. The results demonstrated significant enrichment in mammosphere forming capacity in the lipid droplet^hi^ population (Figure 3d). Taken together, there is strong evidence that lipid droplet enrichment correlates with CSC traits in the BT474 breast cancer cell line.

### 3.4. Targeting Lipid Metabolism Directly Impact CSCs

**Figure 4:**
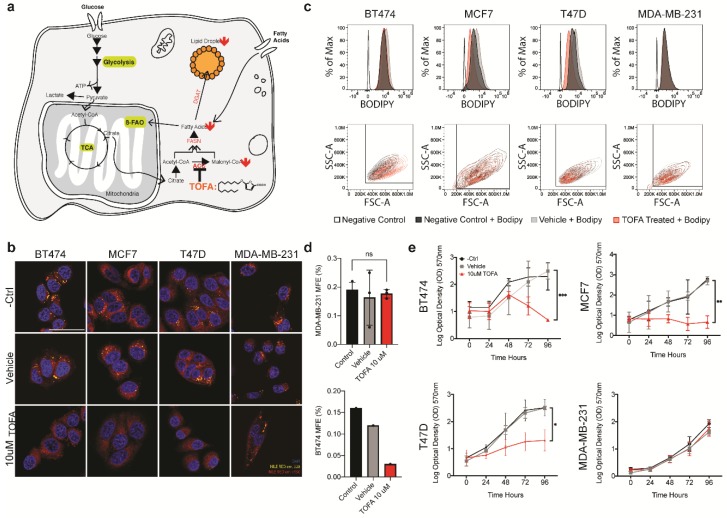
TOFA treatment reveals lipid metabolic addiction in a panel of breast cancer cell lines. (**a**) Schematic depicting the effects of TOFA treatment on lipid metabolism. (**b**) Confocal microscopy of lipid droplets in the cell lines used in this study. Lipid droplets were visualized for control, vehicle, and following 48 h of 10 μM TOFA treated with Nile Red. The scale bar represents 50 μm. (**c**) FACS profile of cells treated with media, vehicle or TOFA for 48 h. From top to bottom, BODIPYTM signal across the treatment groups, side scatter vs. forward scatter plots across the treatment groups. (**d**) Second generation mammosphere forming efficiency for MDA-MB-231 and BT474 following pre-treatment with TOFA, vehicle or regular growth media (*n* = 2). (**e**) Growth of each cell line over time in the presence of 10 μM TOFA, vehicle or growth media. Significance was calculated using the two-tailed *t*-test. * *p* < 0.05, ** *p* < 0.01, *** *p* < 0.001.

Having observed an enhancement in the stem cell pool in lipid droplet^hi^ populations, we reasoned that if we were able to effectively target this population, we should affect the overall fitness of the cell lines. In order to target the metabolic pathways that lead to the accumulation of lipid droplets, we choose to use 5-tetradecyloxy-2-furoic acid (TOFA), an inhibitor of acetyl-CoA carboxylase-α(ACCA). Through inhibition of ACCA, the rate-limiting enzyme in long fatty acid biosynthesis, TOFA has been shown to effectively block fatty acid biosynthesis, therefore limiting FA and lipid droplet accumulation (Figure 4a and [30]). Therefore, we asked whether exposure to TOFA would impact lipid droplet level, cellular proliferation, and/or stemness in our cell line panel.

We began by evaluating the effect of TOFA on lipid metabolism and total lipid droplet number in our panel of cell lines. First, we evaluated the effects of TOFA treatment on FAO. With the exception of MDA-MB-231, we found a significant decrease in FAO following 24 h of TOFA treatment (Figure S4a). This could be attributed to the observed decrease in lipid droplets that were detected in the cells following treatment with 10 uM TOFA (Figure 4b). For a more quantitative evaluation of the effect of TOFA on the lipid droplet number across our cell lines, we used our previously established BODIPY FACS protocol. This analysis confirmed the efficacy of TOFA in reducing the lipid droplets in the cells post-treatment (Figure 4c). In line with the FAO and imaging data, MDA-MB-231 did not show a significant reduction in lipid droplets following TOFA treatment, suggesting that MDA-MB-231 is not reliant upon de novo fatty acid biosynthesis to meet its fatty acid requirements. Interestingly, BT474 did not have a significant shift in BODIPY^TM^ signal following sorting. This was unexpected given the clear differences seen both in the FAO levels and in the imaging. Despite the lack of change in the overall BODIPY^TM^ signal, we noticed a shift in cellular distribution in the SSC/FSC plots, which suggested an increase in cell size following treatment. This increase in cell size, with the accompanying increase in BODIPY^TM^ labeled cellular membranes, could account for the lack of difference observed in the histograms.

Having demonstrated that lipid droplet containing populations were enriched in CSCs, we asked whether treatment with TOFA was sufficient to deplete the CSC populations in the cell lines that were shown to respond to treatment. In order to address this, we performed a qPCR analysis on a panel of stem cell-associated genes in control and treated populations. Despite the global effects on lipid droplet number observed for BT474, T47D and MCF7, TOFA treatment only had marginal effects on the expression of stemness related genes in MCF7 and T47D, while affecting BT474 more significantly (Figure S4b). For a functional validation of these observations, we evaluated mammosphere forming efficiency for both BT474 and MDA-MB-231 for the following conditions: control, vehicle-treated and TOFA treated. Following 5 days of acute TOFA treatment, the surviving populations of BT474 and MDA-MB-231 were washed in TOFA-free growth media to remove any residual TOFA. They were then singularized and seeded in low adhesion, low differentiation conditions in the absence of TOFA and evaluated for mammosphere formation efficiency (Figure 4d and Figure S4c). In line with the qPCR data and imaging data, we observed a significant decrease in second-generation mammosphere-forming capacity for BT474, while MDA-MB-231 had no marked effect on mammosphere generation following TOFA treatment.

The observation that TOFA acts as a potent inhibitor of lipid metabolism and stemness in a subset of cell lines prompted us to evaluate its impact upon proliferation. To do this, we monitored cell growth for 96 h across three treatment groups: growth media, vehicle, and 10 uM TOFA. With the exception of MDA-MB-231, TOFA treatment had a significant impact on the growth of all the cell lines in our panel, severely impeding proliferation (Figure 4d). Although the mammosphere formation assay suggested that TOFA negatively affected the stem cell pool in BT474, these results suggest a more global addiction to the ACCA-mediated fatty acid metabolism that may extend beyond the stem cell pool in these lines.

## 4. Discussion

The resurgence of interest in tumor metabolism has largely focused on the dysregulation of metabolic pathways in the primary tumor itself. However, there is increasing evidence that the metabolic hallmarks of singular populations could play a role in determining their response to therapeutic challenges. Indeed, over the past five years, numerous reports have emerged suggesting that alterations in lipid metabolism correlate with resistance [24,31]. The mechanisms by which dysregulated lipid metabolism contribute to resistance still remain largely unknown. In this study, we investigated the relationship between lipid metabolism and stemness traits by interrogating a panel of breast cancer cell lines.

One of the hallmarks of therapeutic resistant populations is an observed increase in cytoplasmic lipid droplets. Lipid droplets are the lipid storage organelle of the cell, in which neutral lipids such as triglycerides and sterol esters are sequestered. They are dynamic organelles that arise from pools of lipids within the ER in response to stress or alterations in metabolism. The first association of lipid droplets with tumor cells was first made over 50 years ago [32]. Recently, lipid droplets have been shown to correlate with stemness in colorectal cancer [33]. Despite that, surprisingly few have focused on the use of the lipid droplet as a biomarker in breast cancer.

The protein component of lipid droplets could be used as a proxy and facilitate their inclusion in histopathological analyses. In this study, we have focused on PLIN2. PLIN2 is a member of the perilipin family of proteins that includes PLIN2, -3, -4, and -5. This family of lipid-droplet associated proteins plays an instrumental role in lipid droplet formation, stability and trafficking [19,34]. Querying the Kaplan–Meier plotter, we observed a significant negative correlation between lipid droplet associated protein PLIN2 expression level and relapse-free survival in breast cancer patients [23]. This observation stimulated us to better understand the association. One of the outstanding questions in the field is whether the lipid droplets that have been observed in therapeutic resistant cell populations are the result of de novo synthesis in the face of a therapeutic challenge, or if they are an enrichment of a previously metabolically distinct population of cells. The finding that PLIN2 expression in the primary tumor correlates with a worse prognosis suggests that at least in a subset of patients, pre-existing alterations in lipid metabolism could correlate with disease progression.

Interestingly, evaluation of a panel of breast cancer cell lines revealed distinct differences in lipid droplet number between the cell lines. This is despite being maintained in similar media conditions, at comparable confluency. It is interesting to note, as beautifully cataloged in a recent study, that despite having been maintained for 40-plus years in culture, in non-physiological conditions, the metabolic profiles of these cell lines have retained some significant differences, suggesting perhaps a metabolic addiction [35]. In this regard, it is important to highlight that even within individual cell lines, we observed a high degree of heterogeneity in terms of lipid droplet loading within the population. This variation could reflect alterations in the cell cycle, or intriguingly, it could be a marker of metabolically distinct clonal populations, among which CSCs within the cell lines themselves.

One of the most elusive, yet highly sought-after contributors to heterogeneity is the population of CSCs. The CSC theory of tumor progression proposes that it is this population that resists therapeutic intervention, and acts as the seed for tumor recurrence. Therefore, efficient targeting of this population would lead to higher therapeutic efficacy and better long-term survival rates. However, for many tumor types, we still lack robust markers to enable the identification and characterization of the CSC population. Interestingly, we observed a strong correlation between lipid droplet number and stemness in the cell lines we used in this study.

Having devised a method to sort the population based upon lipid droplet number, we were able to demonstrate an increase in mammosphere forming capacity and stem cell markers in the lipid droplet^hi^ segment of the population. An additional characteristic of lipid droplet containing cells which emerged from this study was the correlation between lipid droplets and increased cytosolic complexity, which was detected as increased SSC at FACS. The demonstration that lipid droplets contribute to increased side scatter provides an interesting stain-free mechanism to distinguish lipid droplet^hi^ populations. Taken together, this data suggests that inclusion of lipid droplets into the current criteria for CSC identification could provide an additional, low-cost parameter for stem cell identification.

We also demonstrate that this feature of CSC metabolism can be exploited in therapeutic settings by targeting lipid metabolism. In line with previous reports for CRC, exposure to TOFA, an ACAA targeting drug, profoundly impacted upon lipid droplet persistence in our panel of cell lines [36]. Treatment with TOFA severely affected proliferation in three out of the four lines tested. In the case of BT474, for which we have shown a strong correlation between lipid droplet abundance and stemness, TOFA mediated lipid droplet depletion also resulted in a decrease in the expression of several markers of stemness. An interesting observation for further studies is the cell line MDA-MB-231 that appeared to be largely resistant to treatment by TOFA, as seen by the lack of impact upon lipid droplets number as quantified at FACS. In line with this, we observed no effect of TOFA treatment on viability or stemness in the MDA-MB-231, despite observing a drastic effect in all other cell lines. This data suggests that MDA-MB-231 is able to fulfill its fatty acid demands by other means. As TOFA specifically targets de novo fatty biosynthesis, it would be informative to perform a more inclusive study, using a panel of inhibitors targeting the various druggable components of the lipid droplet assembly cascade. However, despite the lack of efficacy on MDA-MB-231, the efficacy of TOFA in inhibiting proliferation of the other cell lines used in this study suggests that it would be interesting to explore the inclusion of lipid metabolic targeting compounds into current therapeutic regimes.

## 5. Conclusions

In this study we demonstrated a correlation between lipid droplet number and stemness features in a panel of breast cancer cell lines. Yet, our observations were limited to steady-state growth conditions. There is ample literature addressing the induction of lipid droplet accumulation in response to cellular stressors, including chemotherapeutic administration. Therefore, it would be critical to understand if and how lipid droplet biosynthesis is dysregulated following therapeutic exposure. For example, do lipid droplets accumulate in previously deplete populations? If so, what are the stress response pathways governing the expansion? Are lipid droplets dispensable for survival? It is our hope that the answers to these questions will reveal novel lipid metabolic targets in malignancy specific pathways.

## Figures and Tables

**Table 1:** Molecular classification of breast cancer cell lines.

Classification	ER	PR	HER2	Example Cell Lines [26,27]
Luminal A	+	+/-	-	**MCF7**, **T47D**, SUM185
Luminal B	+	+/-	+	**BT474**, MDA-MB-361
Claudin-low	-	-	-	**MDA-MB-231**, BT549
Basal	-	-	-	MDA-MB-468, SUM 190
HER2	-	-	+	SKBR3, MDA-MB-453

ER, estrogen receptor; PR, progesterone receptor; HER2, human epidermal growth factor receptor 2. The cell lines indicated in bold were used in this study.

## Supplementary Materials

The following are available online at https://www.mdpi.com/2077-0383/9/1/87/s1, Figure S1: Lipid droplets correlate with stemness in breast cancer cell lines, Figure S2: BT474 and T47D cell lines internalize fatty acids and are able to form lipid droplets in a dose dependent manner, Figure S3: Gating Strategy for CD44hi/CD24low in BT474 and MDA-MB-231 cell lines, Figure S4: Inhibition of de novo fatty acid biosynthesis effects stemness traits in BT474.
