# Supplementary material for: Lipid Droplets Define a Sub-Population of Breast Cancer Stem Cells
Source: J Clin Med. 2019 Dec 29;9(1):87. doi: 10.3390/jcm9010087 (PMC7019257; doi:10.3390/jcm9010087)
Supplement: Supplementary file 1 [file jcm-09-00087-s001.pdf]

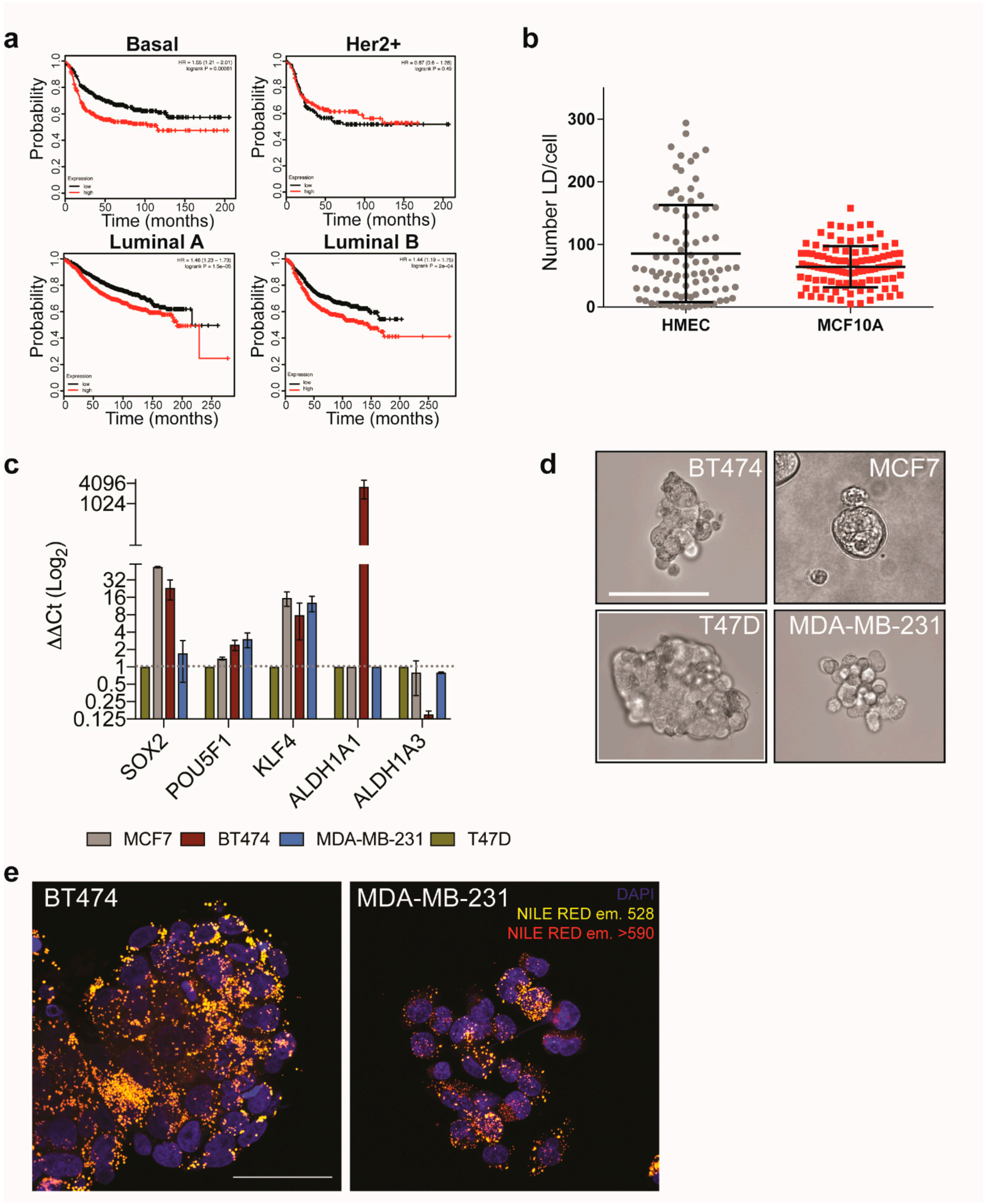

Supplementary Figure S1. Lipid droplet accumulation correlates with stemness in breast cancer cell lines. **(a)** Correlation between PLIN2 levels and Relapse Free Survival for the four main breast cancer histotypes. **(b)** Quantitative analysis of the lipid droplets content of HMEC and MCF10a cell lines. 100 cells were analyzed for each cell line. Data are represented as mean ±SEM. **(c)** Gene expression normalized to T47D obtained from quantitative PCR analysis of a panel of stemness related genes. **(d)** Representative images of mammospheres obtained in the mammosphere formation assay. Scale bar represents 200 μm **(e)** Maximum projection of mammospheres stained with Nile Red for visualization of lipid droplets. Scale bar represents 50 μm.

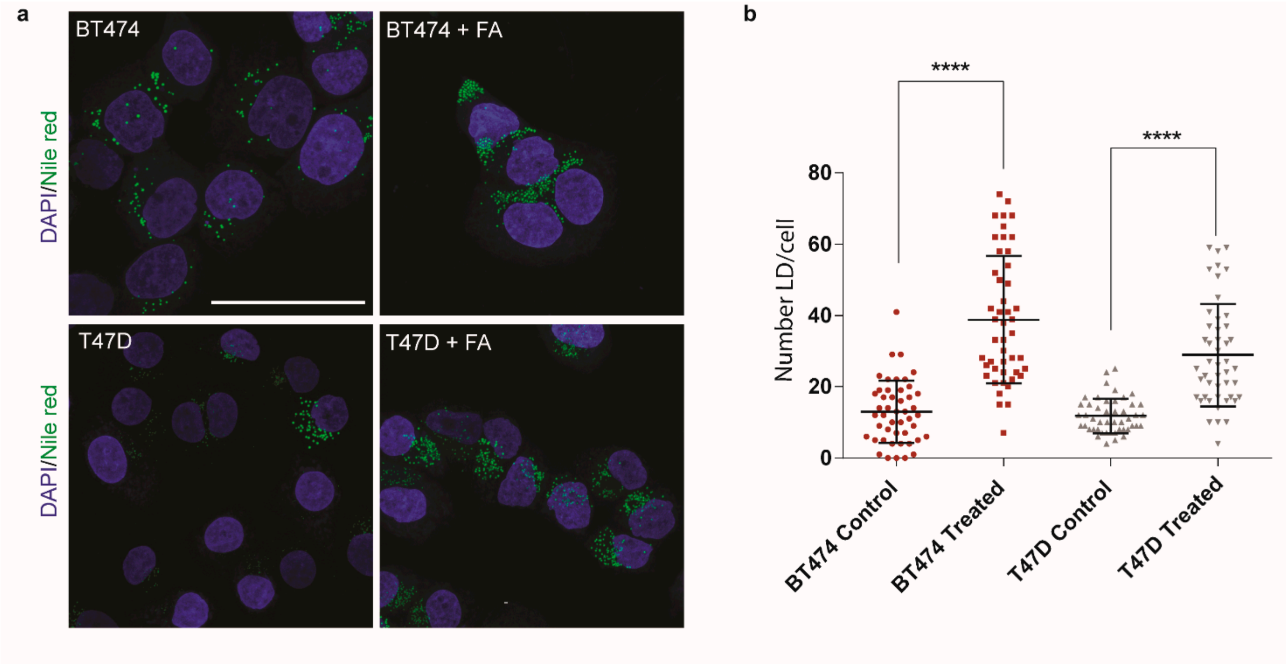

Supplementary Figure S2. BT474 and T47D cell lines internalize fatty acids and ae able to form lipid droplets in a dose dependent manner. **(a)** BT474 and T47D cells were treated with 50 μM Palmitic acid for 72 hours and then fixed and stained with nile red. Confocal images show control cells for the two cell lines. Scale bar represents 50 μm (b) Quantitative analysis of the lipid droplet content. 100 cells were analyzed for each condition. Date are represented as mean ±SEM. Significance was calculated using two-tailed t-tests **** $p < 0.0001$.

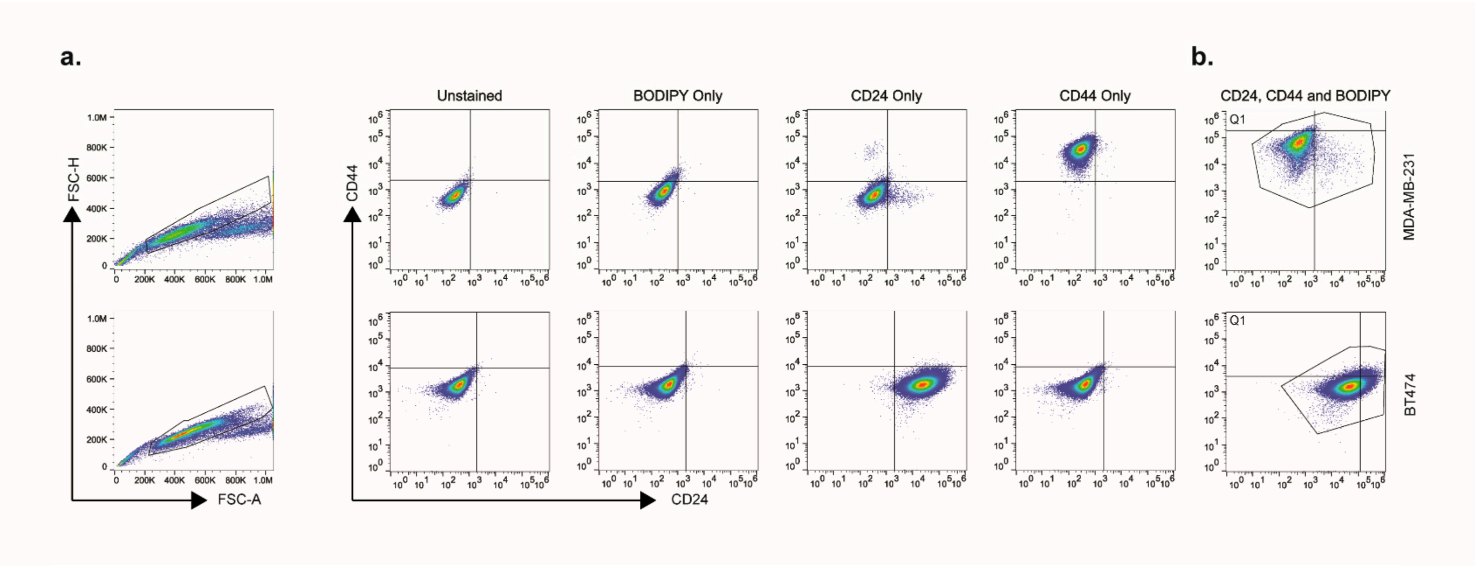

Supplementary Figure S3. Gating strategy for CD44^{hi}/CD24^{lo} in BT474 and MDA-MB-231 cell lines. **(a)** The raw data was first assessed by plotting forward scatter height (FSC-H) and forward scatter area (FSC-A) in order to discriminate cells from debris. The cell lines were then assessed for expression of CD44 and CD24 levels using individual antibodies alone. **(b)** Finally, as depicted in Figure 3a, the CD44, CD24 and BODIPY™ levels were assessed for each cell line. The total cell population was divided into four quadrants with quadrant (Q1), representing the relative CD44^{hi}/CD24^{lo} populations for each cell line. Q1 was than adjusted to contain between 0.5% and 1% of the total population. The level of BODIPY™ in the newly defined CD44^{hi}/CD24^{lo} population subset was then compared to the level of BODIPY™ contained in the total population.

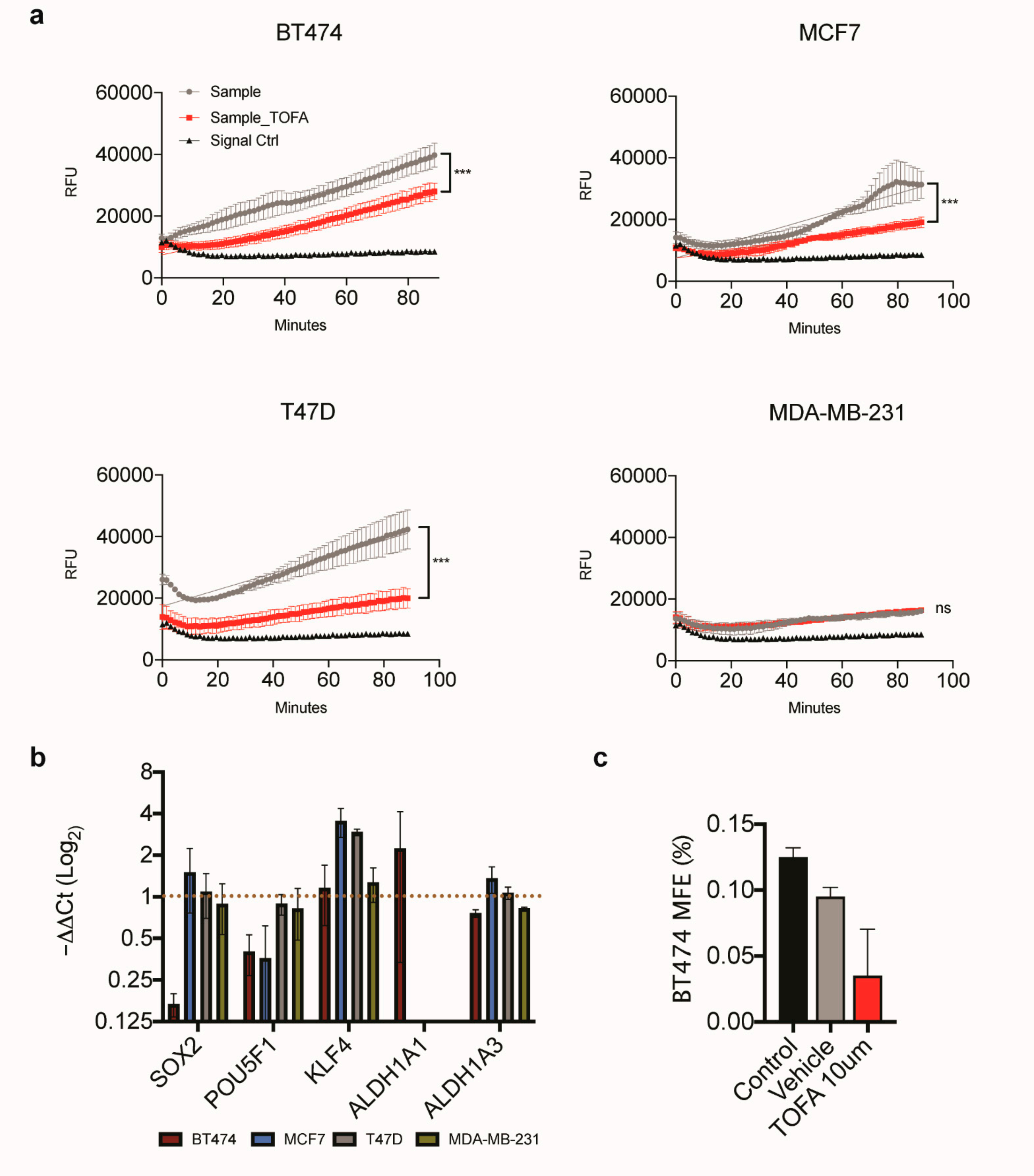

Supplementary Figure S4. Inhibition of de novo fatty acid biosynthesis effects stemness traits in BT474. **(a)** Fatty Acid Oxidation (FAO) rates of BT474, MCF7, T47D and MDA-MB-231. FAO was measured for control and TOFA treated groups. The measurements were made following 24 hours of TOFA treatment. **(b)** qPCR data depicting the fold change of stemness genes in the breast cancer cell panel following treatment for 48 hours with 10 µM TOFA or vehicle. **(c)** Second generation mammosphere forming efficiency of BT474 in the presence of 10 µM TOFA, vehicle or regular growth media (n=2). *** $p < 0.001$
